# HJURP sustains ferroptosis sensitivity of TNBC by interacting with SLC7A11 and maintaining its function

**DOI:** 10.1186/s43556-024-00208-9

**Published:** 2024-10-03

**Authors:** Yongxia Chen, Kaili Cen, Linbo Wang, Jian Ruan, Yunlu Jia

**Affiliations:** 1 Department of Surgical Oncology, Sir Run Run Shaw Hospital, Zhejiang University School of Medicine, Hangzhou, 310020 China; 2 Department of Medical Oncology, the First Affiliated Hospital, Zhejiang University School of Medicine, Hangzhou, 310003 China; 3 Department of Hematology-Oncology, Taizhou Hospital of Zhejiang Province, Taizhou, 317000 Zhejiang China

Dear editors:

Triple-negative breast cancer (TNBC) is characterized by more aggressive phenotype, limited targeted therapies and a higher risk of recurrence, which accounts for about 10–15% of all breast cancers. Various new agents and combination strategies have been explored to further understand molecular and immunological aspects of TNBC [1]. Ferroptosis is a recognized programmed cell death driven by the iron-dependent accumulation of lipid hydroperoxides and reactive oxygen species (ROS), which was involved in the pathogenesis, development as well as therapeutic targets of multiple types of cancer. Studies suggest TNBC may be more susceptible to ferroptosis than other breast cancer types [2]. However, the role of ferroptosis in breast cancer remains complex, necessitating further research for effective treatment strategies.

HJURP (Holliday Junction Recognition Protein), is primarily known for its role in maintaining and functioning mitotic chromatin. As a specific molecular chaperone, HJURP ensures the accurate placement of the histone variant CENP-A at centromeres, which are critical for chromosome segregation during cell division. In cancer research, HJURP has emerged as a significant player due to its dysregulation in various malignancies. Abnormal HJURP expression can disrupt CENP-A distribution, leading to chromosomal instability, a hallmark of cancer development [3]. HJURP also participates in DNA damage repair, particularly in the response to double-strand breaks, safeguarding against mutation accumulation that could drive carcinogenesis. Methylation of HJURP is associated with reduced cell cycle control and enhanced tumor growth, while HJURP is implicated in tumor immune evasion, highlighting its potential as both a diagnostic and therapeutic target in oncology. Previously, by mapping the unique active cis-regulatory landscape in TNBC, we identified the novel enhancer-associated gene-HJURP [4]. Targeting HJURP allows for effective suppression of tumor invasion and attenuating metastasis in p53-mutant TNBC. Here we found the key downstream targets of HJURP regulating TNBC proliferation, which is the impact on ferroptosis susceptibility in TNBC.

The study began by examining HJURP expression and key ferroptosis-related markers in breast cancer samples using TCGA datasets. The results revealed positive correlation of HJURP and ferroptosis-related markers, including SLC7A11, ACSL4 and FTH1, suggesting a potential role for HJURP as a ferroptosis driver gene. We also analyzed the mRNA expression levels of key ferroptosis-related markers in breast cancer cell lines with different HJURP expression levels. Consistent patterns in the expression levels of HJURP and the identified ferroptosis-related markers were identified across breast cancer samples (data not shown here). Additionally, RNA sequencing was employed to assess the mRNA profiling of critical ferroptosis-related genes following HJURP knockdown in MDA-MB-231 cells (Fig. 1a). In comparison with the control group, the analysis of cells with manipulated HJURP expression revealed a significant enrichment of genes involved in the ferroptosis regulatory pathway. These findings collectively suggest a correlation between elevated HJURP expression and increased levels of ferroptosis-related markers, particularly in the triple-negative/basal-like breast cancer subtype. To further confirm the role of HJURP in regulating ferroptosis, we chose relevant breast cancer cell lines for HJURP knockdown assay. The cells were transfected with shRNA to modulate HJURP expression, and subsequent Western blot and qPCR analyses were performed to evaluate the protein and mRNA levels of two key ferroptosis-related markers, SLC7A11 and FTH1 (Fig. 1b). SLC7A11, also known as xCT, is a subunit of a cystine-glutamate antiporter that plays a critical role in maintaining intracellular glutathione levels, which are essential for antioxidant defense and therefore can protect cells from oxidative stress-induced death like ferroptosis. FTH1 (Ferritin Heavy Chain 1) is a crucial protein involved in iron storage and metabolism within cells. It plays a significant role in maintaining iron homeostasis and preventing oxidative stress by sequestering excess iron in a non-toxic ferritin complex. By modulating SLC7A11 and FTH1, HJURP may influence the availability of cystine and consequently glutathione synthesis, thereby affecting the vulnerability of TNBC cells to ferroptotic cell death. We next measure cell viability and cytotoxicity using MTT assays after exposing TNBC cells to ferroptosis inducers (Erastin). Breast cancer cell line (MDA-MB-231) with downregulated HJURP levels exhibited reduced viability and presented more susceptible to ferroptosis (Fig. 1b)**.** As ferroptosis is characterized by lipid peroxidation, we performed lipid peroxidation assays and measured malondialdehyde (MDA) levels, and cells with downregulated HJURP levels have lower glutathione (GSH) levels and increased susceptibility to ferroptosis (Fig. 1c).Based on these results, we further provide experimental evidence supporting the role of HJURP in modulating the expression of key ferroptosis-related genes, shedding light on the regulatory mechanisms involved in ferroptosis of TNBC. To investigate the direct or indirect regulatory pathway of HJURP and ferroptosis-promoting factors, we perform in silico protein-protein interaction prediction using bioinformatics tools to identify potential interaction. Co-IP experiments were conducted to determine whether HJURP physically interacts with SLC7A11 and FTH1. As a result, detection of SLC7A11 and FTH1 bands in the Western blot of HJURP IP samples indicated a physical interaction between HJURP and these ferroptosis regulators (Fig. 1d). In a more physiological context, we have conducted endogenous co-IP experiments to confirm the interaction between HJURP and SLC7A11 without the use of overexpression constructs in MDA-MB-231 cells. Finally, we investigated the functional consequences of the interaction, and assessed whether the interaction between HJURP, SLC7A11 and FTH1 has any impact on cellular processes related to ferroptosis susceptibility. Rescue functional experiments was conducted, and we co-transfected HJURP-manipulated cells with ferroptosis-related SLC7A11 overexpression vector, to determine whether HJURP-driven ferroptosis was altered when SLC7A11 expression is manipulated. As a result, we observed restoring SLC7A11 expression rescues HJURP-driven ferroptosis (data not shown here). To explore the impact of HJURP on prognosis and treatment response in breast cancer patients, we conducted survival analysis using the TCGA database. Our preliminary results revealed that higher HJURP expression correlates with poorer overall survival (OS) outcomes. Furthermore, we investigated whether certain treatment panels may benefit patients with different levels of HJURP expression. Our analysis suggested that patients with lower HJURP expression may respond better to chemotherapy. These findings provide initial evidence of the potential clinical significance of HJURP in breast cancer prognosis and treatment response. Additional clinical validation studies are warranted to confirm these associations and to further explore the therapeutic implications of HJURP modulation in breast cancer.

**Fig. 1:**
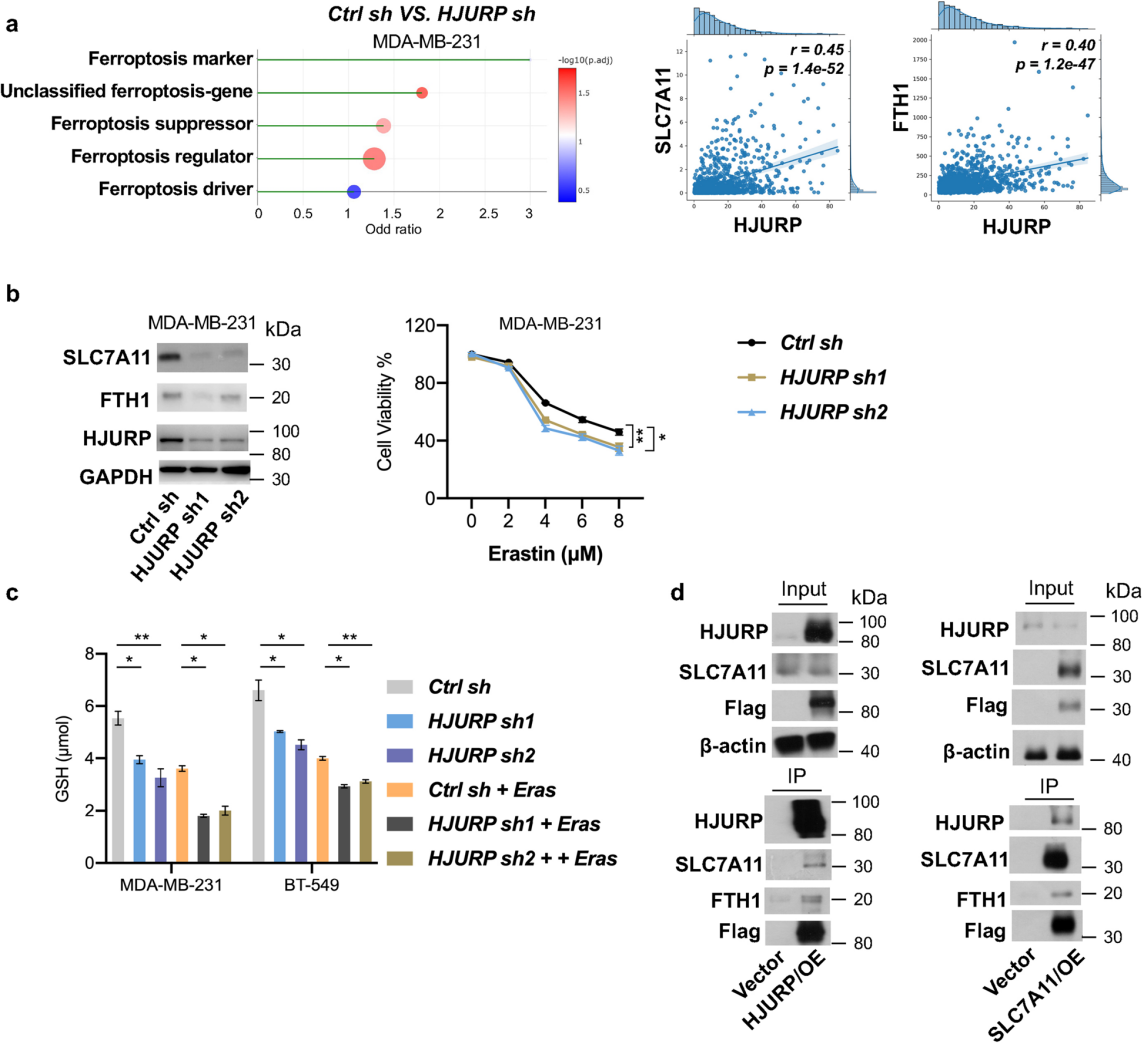
Correlation of HJURP expression and ferroptosis activity in breast cancer, **a** RNA sequencing analysis of critical ferroptosis-related genes following HJURP knockdown in MDA-MB-231 cell line, indicating changes in mRNA profiles (Left) .Positive correlation of HJURP and ferroptosis-related markers (SLC7A11 and FTH1) in breast cancer samples (Right). **b **Western blot analysis of SLC7A11 and FTH1 protein levels in breast cancer cell line (MDA-MB-231) after HJURP knockdown. GAPDH was used as a loading control (Left). MTT assays showing reduced viability and increased susceptibility to ferroptosis in MDA-MB-231 and BT-549 cells with downregulated HJURP, following exposure to the ferroptosis inducer Erastin for 48 h (Right). **c** GSH levels (Right) in breast cancer cell lines (MDA-MB-231 and BT-549) with downregulated HJURP upon treatment with Erastin. **d** Co-IP experiments showing physical interaction between HJURP and ferroptosis regulators SLC7A11 and FTH1 in breast cancer cell lines, detected through Western blot. P values were derived from t-tests to compare a treatment group with a control group. *P < 0.05, ** *P* < 0.01, *** *P* < 0.001

Previously, HJURP is found to be involved in the regulation of ferroptosis in oral squamous cell carcinoma cells, and increased levels of HJURP lead to a reduction in ferroptosis, promoting tumor growth and survival. Here our findings contrast with previous observations in oral squamous cell carcinoma, where HJURP was shown to restrain ferroptosis, suggesting context-specific roles of HJURP in different cancer types [5]. Our study demonstrates HJURP modulates ferroptosis susceptibility through its interaction with SLC7A11 and FTH1 in TNBC, underscoring its potential as a therapeutic target. While the in vitro experiments presented here establish a functional link between HJURP and SLC7A11, further research, including in vivo studies, is needed to fully elucidate the molecular mechanisms underlying this interaction and to confirm the clinical relevance of these findings in TNBC. HJURP expression levels could serve as a predictive biomarker for the response to ferroptosis-inducing therapies in TNBC patients. Patients with higher HJURP levels might benefit more from such treatments. The study supports the exploration of combination strategies involving HJURP-targeted therapies and existing treatments to enhance the sensitivity of TNBC cells to ferroptosis, potentially improving patient outcomes.

## Supplementary Information

Supplementary Material 1.

Supplementary Material 2.

## Data Availability

All data supporting the findings of this study are available within the paper and its Supplementary Information.
