# Supplementary material for: HJURP sustains ferroptosis sensitivity of TNBC by interacting with SLC7A11 and maintaining its function
Source: Mol Biomed. 2024 Oct 3;5:41. doi: 10.1186/s43556-024-00208-9 (PMC11447182; doi:10.1186/s43556-024-00208-9)

Figure 1b

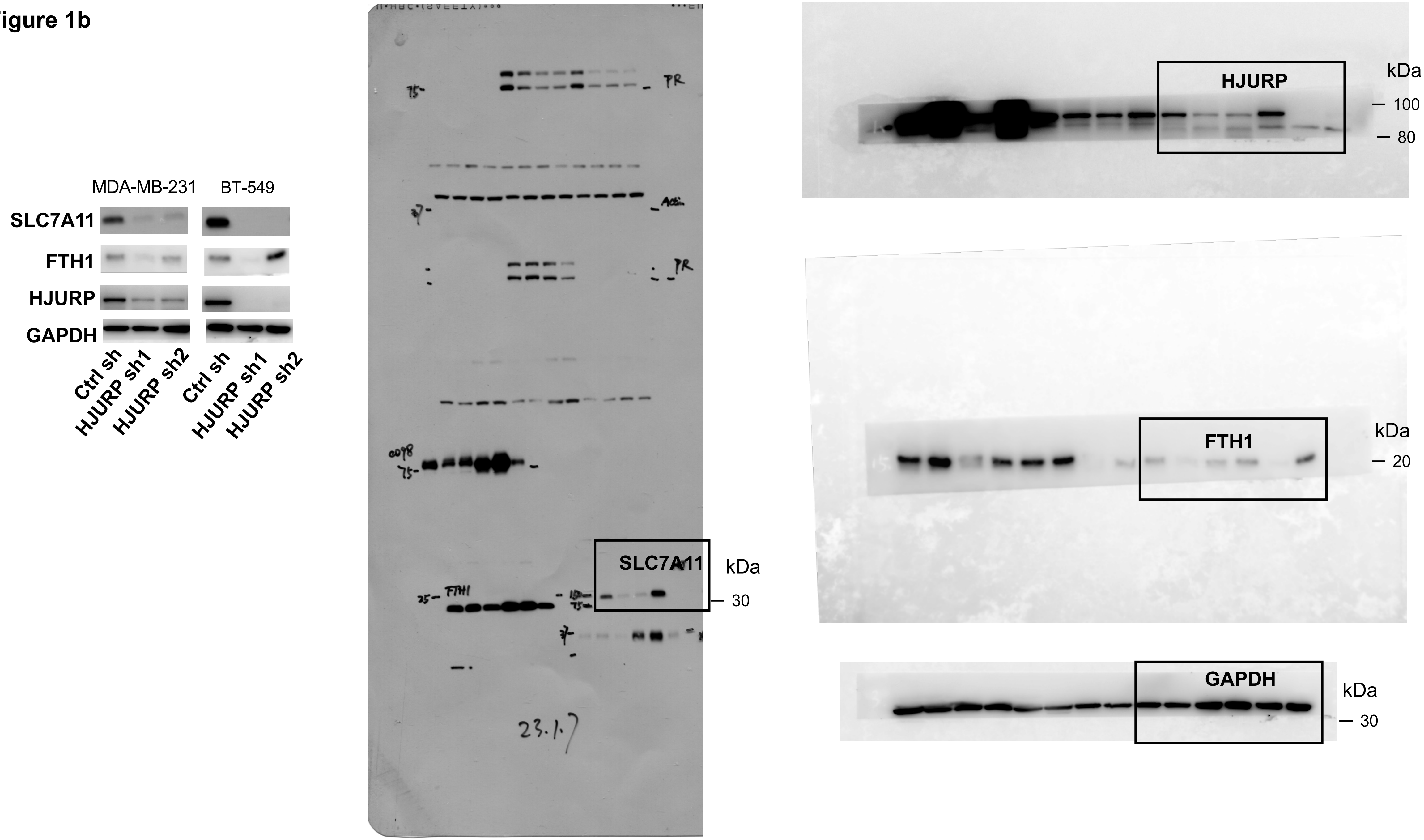
MDA-MB-231
BT-549
SLC7A11
FTH1
HJURP
GAPDH
Ctrl sh
HJURP sh1
HJURP sh2
Ctrl sh
HJURP sh1
HJURP sh2
PR
Actin
FTH1
23.1.7
SLC7A11
kDa
30
HJURP
kDa
100
80
FTH1
kDa
20
GAPDH
kDa
30

Figure 1d (Left)

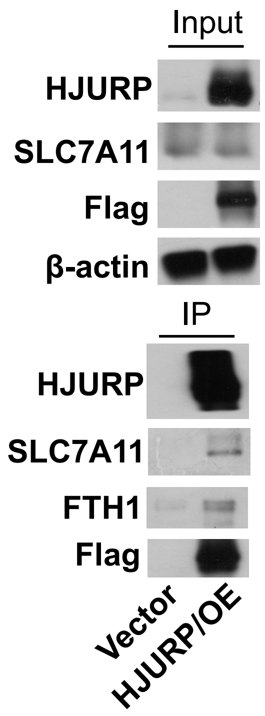

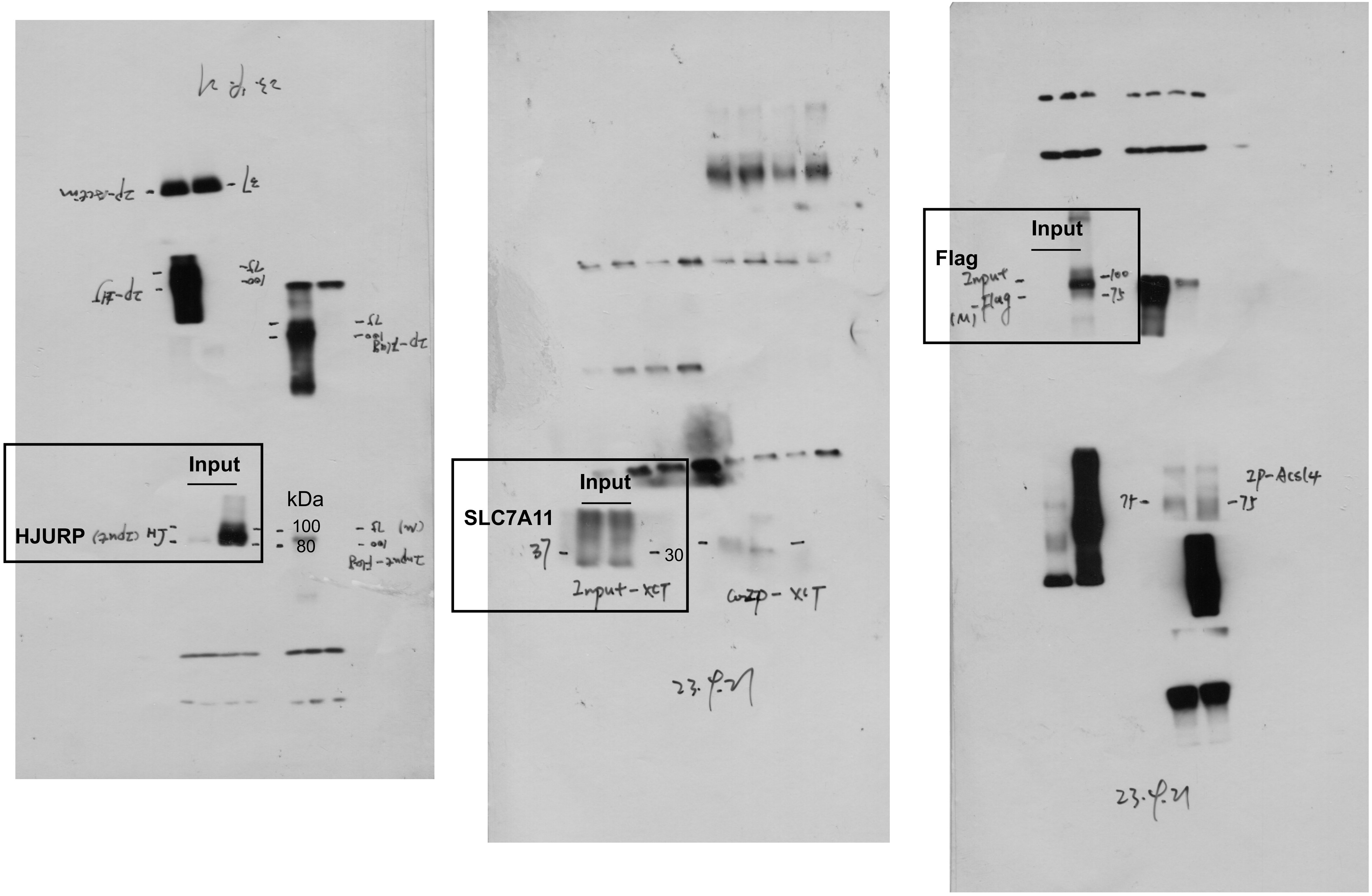

Figure 1d (Left) (Continued)

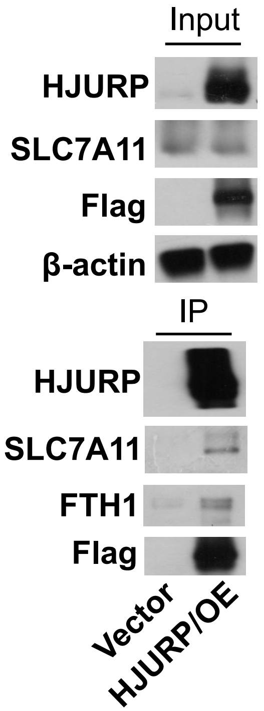

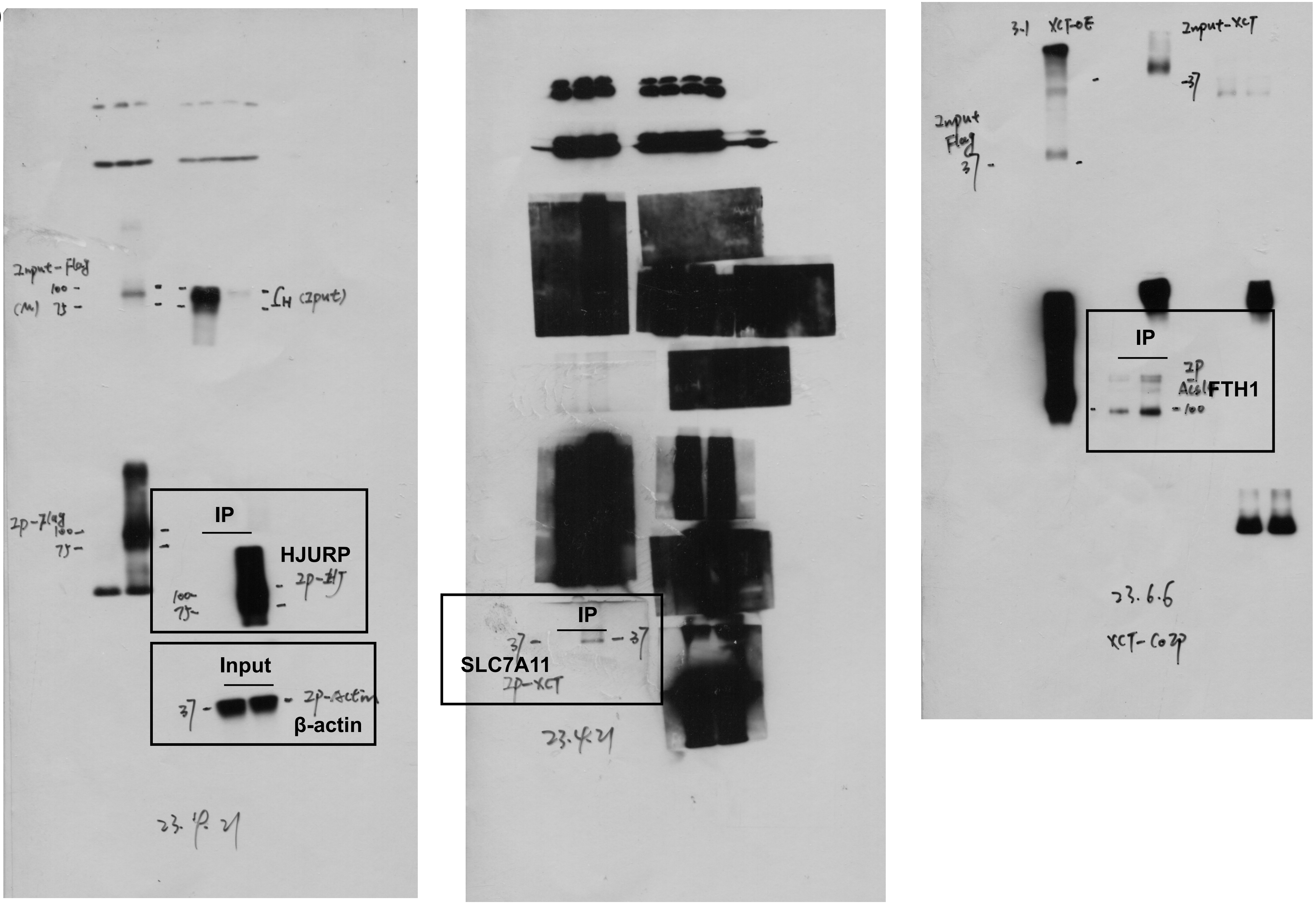

Figure 1d (Left) (Continued)

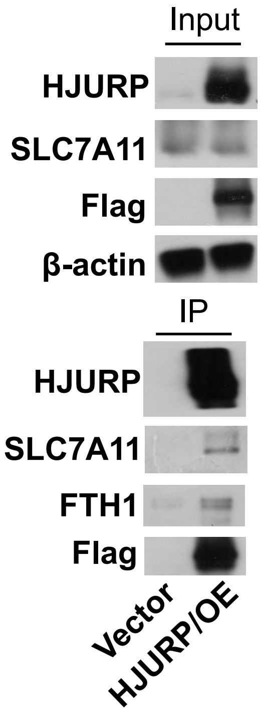

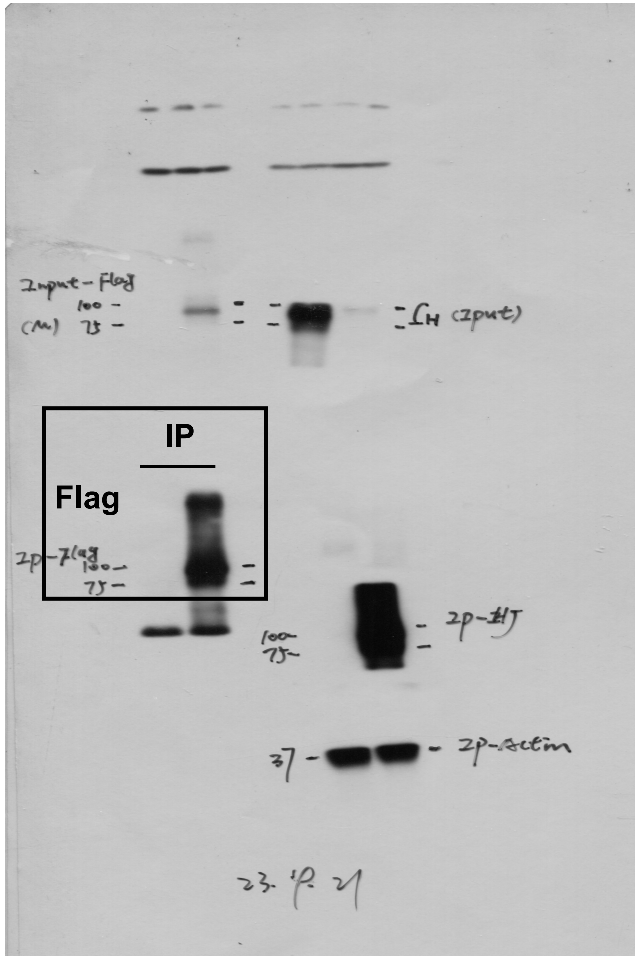

Figure 1d (Right)

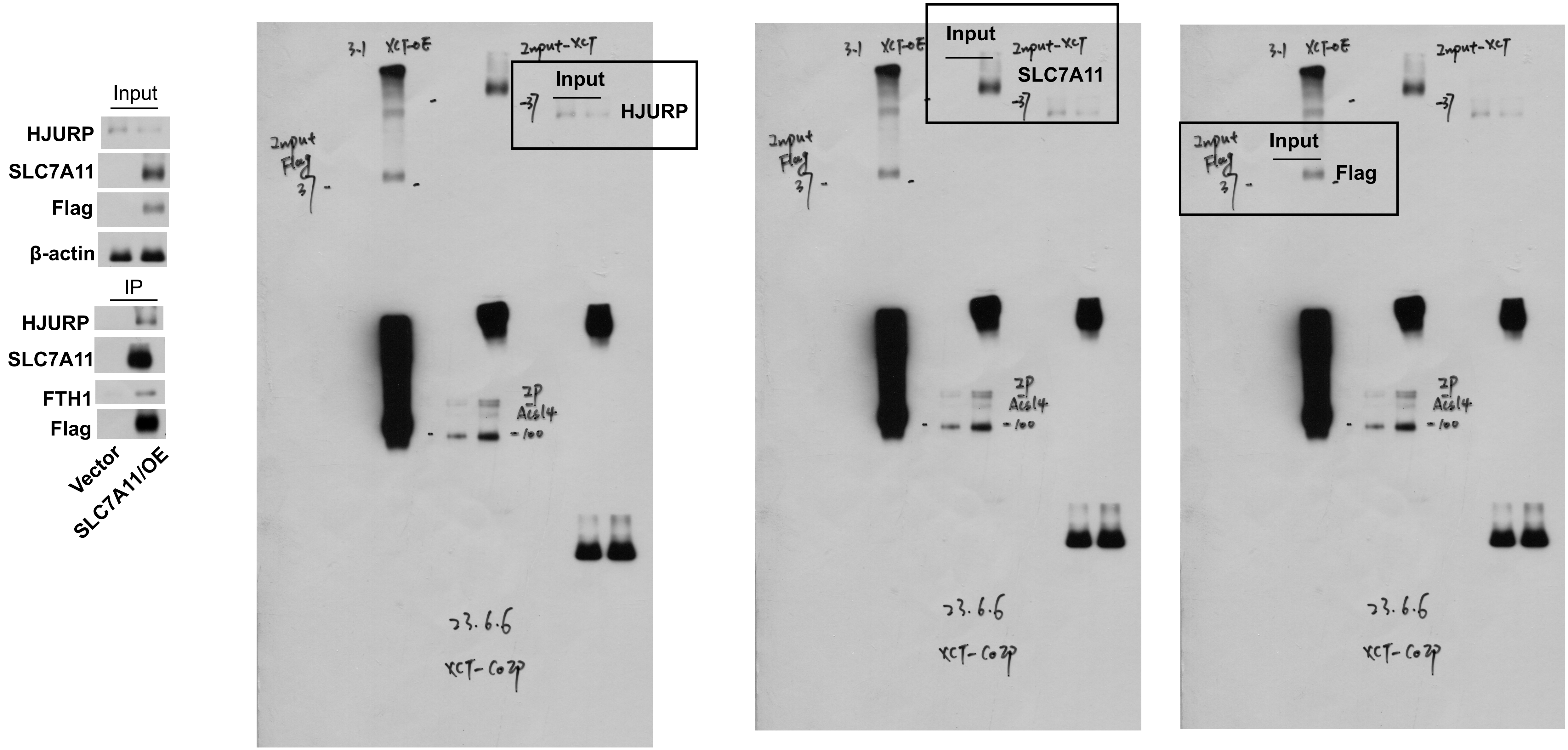

Figure 1d (Right) (Continued)

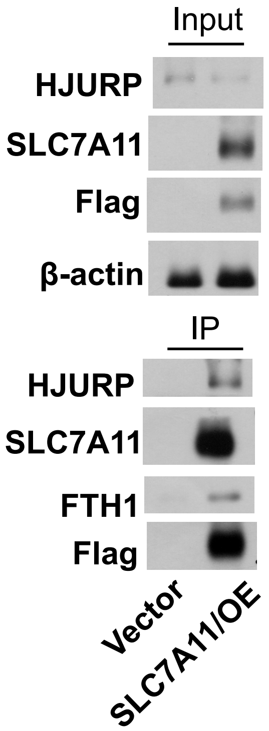

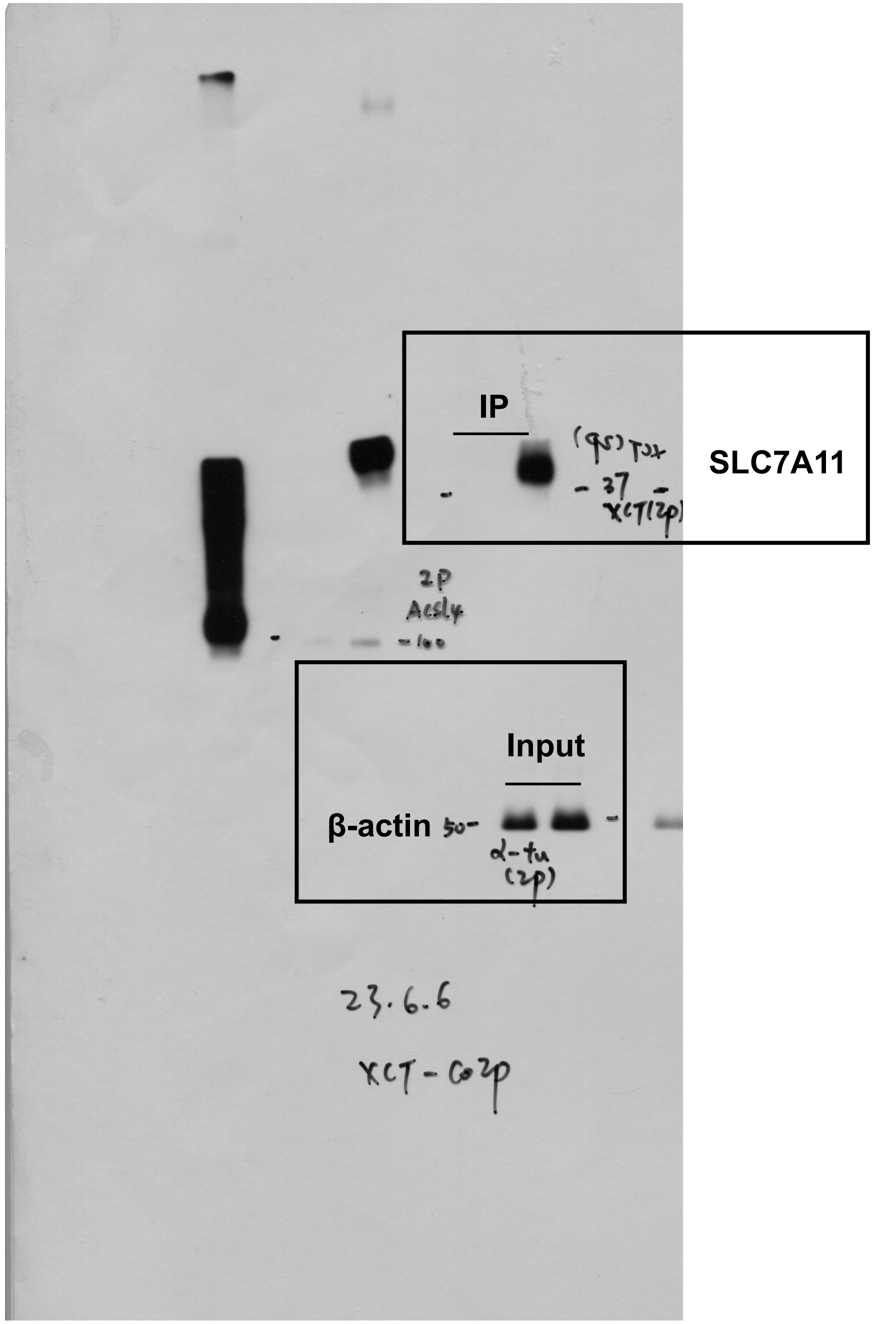

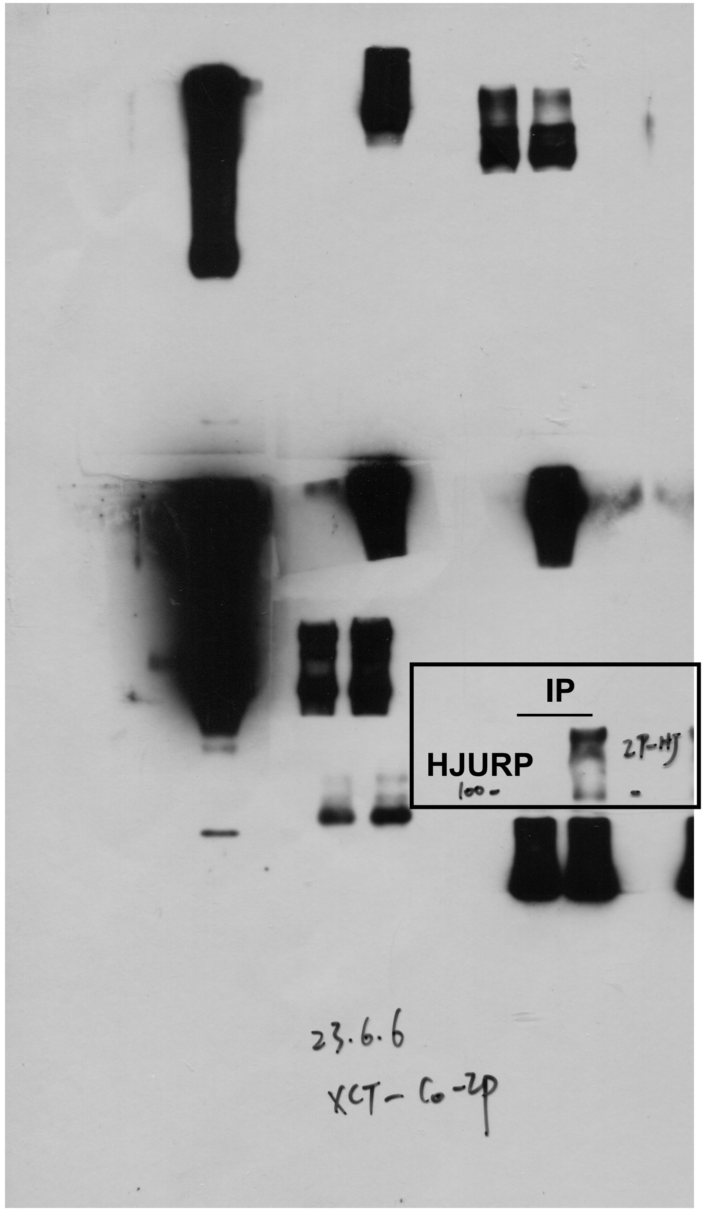

Figure 1d (Right) (Continued)

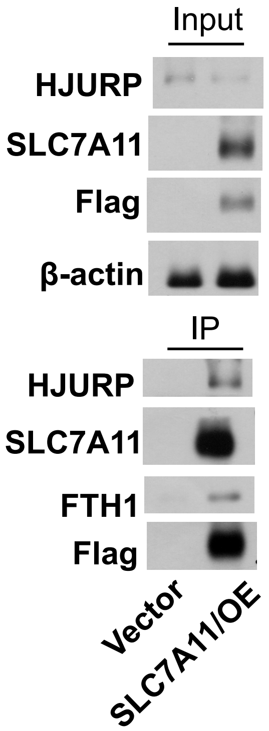

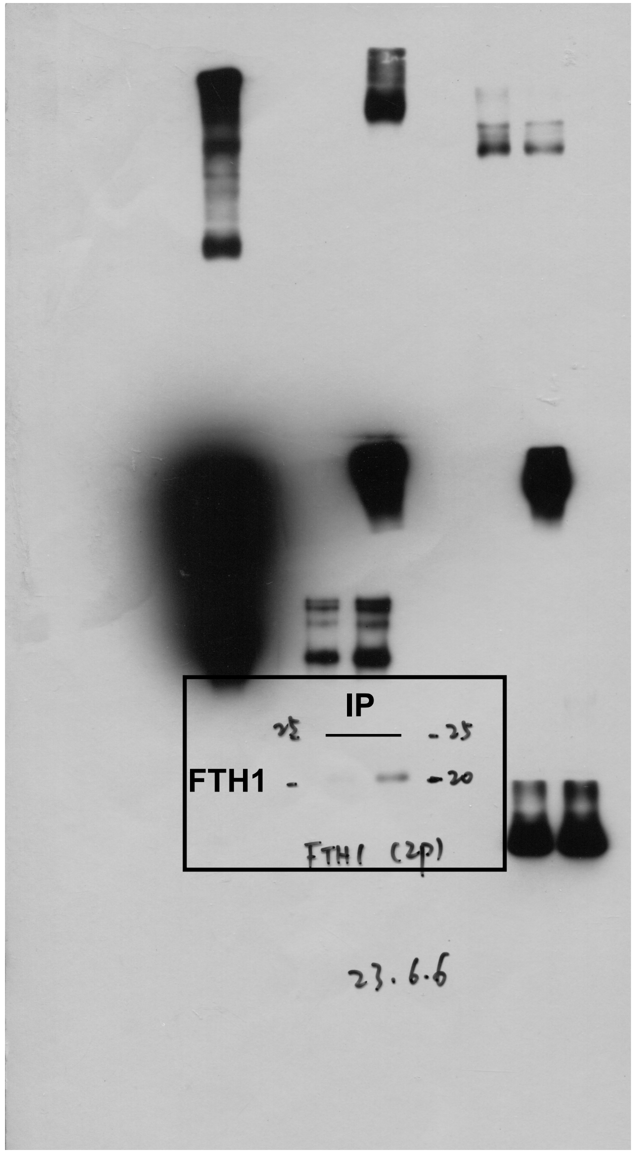

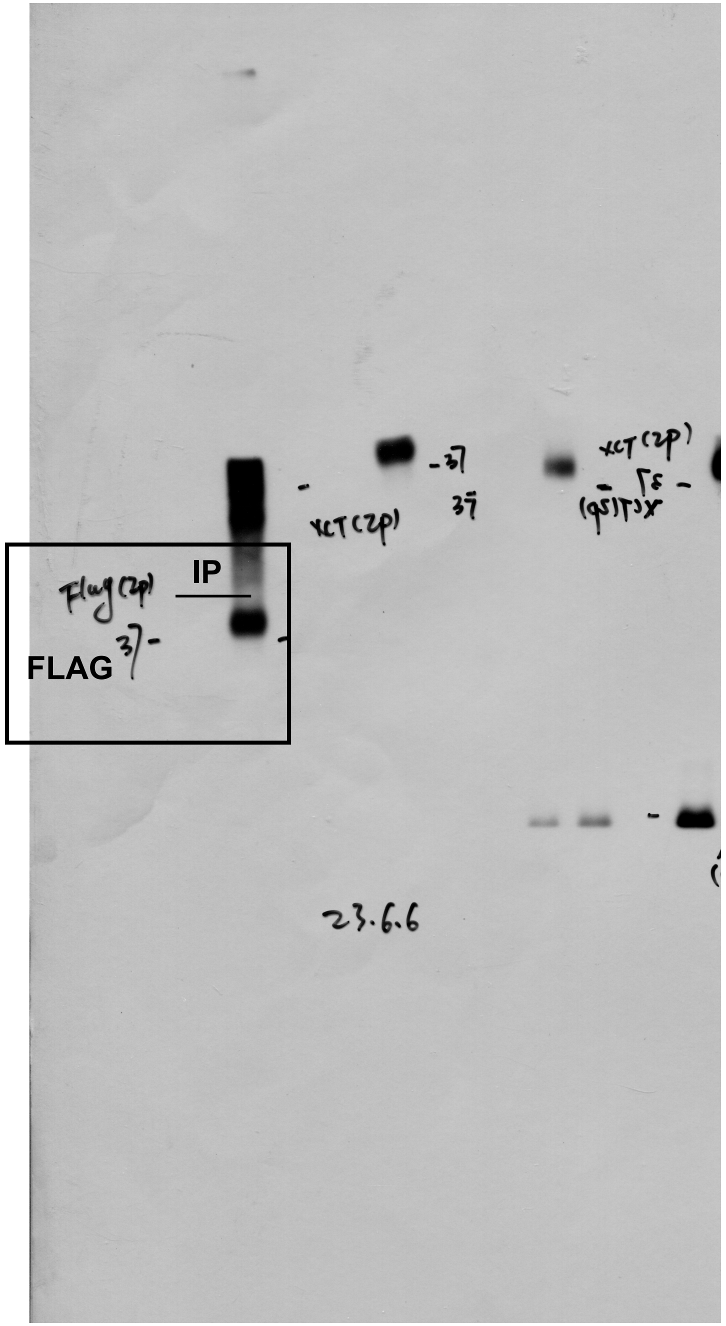

Supplement: Supplementary file 1 — Supplementary Material 1. [file 43556_2024_208_MOESM1_ESM.pdf]
