# Supplementary material for: HJURP sustains ferroptosis sensitivity of TNBC by interacting with SLC7A11 and maintaining its function
Source: Mol Biomed. 2024 Oct 3;5:41. doi: 10.1186/s43556-024-00208-9 (PMC11447182; doi:10.1186/s43556-024-00208-9)
Supplement: Supplementary file 2 — Supplementary Material 2. [file 43556_2024_208_MOESM2_ESM.docx]

**HJURP Sustains Ferroptosis Sensitivity of TNBC by Interacting with SLC7A11 and Maintaining its Function**

Yongxia Chen^1#^, Kaili Cen^3#^, Linbo Wang^1*^, Jian Ruan^2*^ , Yunlu Jia^2*^

*^1^Department of Surgical Oncology, Sir Run Run Shaw Hospital, Zhejiang University School of Medicine, Hangzhou, China,* *310020. ^2^Department of Medical Oncology, the First Affiliated Hospital, Zhejiang University School of Medicine, Hangzhou, China, 310003.*

*^3^Department of Hematology-Oncology, Taizhou Hospital of Zhejiang Province, Taizhou, Zhejiang, China 317000.*

***Corresponding author:**

Linbo Wang, Department of Surgical Oncology, Sir Run Run Shaw Hospital, Hangzhou, China. E-mail address: linbowang@zju.edu.cn.

Jian Ruan, Department of Medical Oncology, the First Affiliated Hospital, Zhejiang University School of Medicine, Hangzhou, China. E-mail address: software233@zju.edu.cn.

Yunlu Jia, Department of Medical Oncology, the First Affiliated Hospital, Zhejiang University School of Medicine, Hangzhou, China. E-mail address: jiayunlu@zju.edu.cn

**Running Title:** Influence of HJURP on Ferroptosis Susceptibility in TNBC

Equal Contribution Note: Yongxia Chen and Kaili Cen contributed equally to this work.

**Methods and Materials**

**Cell lines and Reagents**

Human breast cancer cell lines BT-549 and MDA-MB-231 were mycoplasma-free and were authenticated using short tandem repeat (STR) genotyping within the last 3 years and were verified to be identical with the STR profile in reference databases (CLS Cell Lines Service GmbH). Cells were cultured in Dulbecco’s Modified Eagle Medium (DMEM) with high glucose (D 6429) supplemented with 10% FBS and 19 PEST (Penicillin and streptomycin antibiotics) at 37 °C and 5% CO^2^ under humidified conditions.

**Quantitative RT-PCR**

The RNeasy mini kit (Qiagen) was utilized to extract total RNA, and 1 μg aliquots were employed for the synthesis of cDNA via the qScript™ cDNA Synthesis Kit (Quanta Biosciences). PCR amplification of the cDNA templates was performed on the CFX96 qPCR System (Biorad). GAPDH served as the reference for normalizing the expression of each gene, which was quantified using the 2−delta(Ct) method. The primers used in quantitative RT-PCR are listed below:

|  | ***Fwd*** | ***Rev*** |
| --- | --- | --- |
| ***GAPDH*** | *GTCTCCTCTGACTTCAACAGCG* | *ACCACCCTGTTGCTGTAGCCAA* |
| ***HJURP*** | *CAGACCAGGAAGAGTCAGTTGC* | *CTTCCAGCTCTGTTACCTGCAC* |
| ***SLC7A11*** | *TCCTGCTTTGGCTCCATGAACG* | *AGAGGAGTGTGCTTGCGGACAT* |
| ***FTH1*** | *TGAAGCTGCAGAACCAACGAGG* | *GCACACTCCATTGCATTCAGCC* |

## **Immunoblotting assay**

The cells were lysed using RIPA lysis and extraction buffer (Thermo Fisher Scientific), enriched with proteinase and phosphatase inhibitor cocktails (Roche), for a duration of 30 minutes on ice. Protein amounts were quantified through the Bradford assay. SDS-PAGE preceded immunoblotting, which was followed by the transfer of proteins to a PVDF membrane (Bio-Rad). Primary antibodies were incubated in a cold room overnight, while secondary antibodies were incubated for an hour at room temperature. We used the following antibodies: GAPDH (1: 500, SANTA CRUZ BIOTECHNOLOGY, sc-47724), β-Actin (1:1000, Cell Signaling Technology, #4970), HJURP (1:1000, Sigma Aldrich, HPA008436), SLC7A11 (1:1000, Cell Signaling Technology, #98051), FTH1 (1:500, Cell Signaling Technology, #4393), and GPX4 (1:500, Cell Signaling Technology, #52455). Signals were detected using SuperSignal reagents (Thermo Fisher Scientific) on an ImageQuant LAS500 (GEHealthcare) device.

**Plasmids and transfection**

The two-independent HJURP shRNA vectors and scrambled shRNA were purchased from Sigma-Aldrich - Mission TRC shRNA library. Transfection of the plasmids into breast cancer cell lines was conducted using the Lipo- fectamine 3000 (Invitrogen) transfection reagents follow- ing the manufacturer’s instructions. Cells were harvested for total RNA and protein extraction 48 h after transfec- tion and processed for functional assays.

**Cell proliferation assay**

Cells were seeded at 1.25 × 10^5^ cells per well in 12-well plates. MTT solution (5 mg mL−1 (3-(4, 5-dimethylthiazolyl-2)-2, 5-diphenyltetrazolium bromide) was added to the cells and incubated at 37°C in 5% CO^2^ for 1 h. The cells were resuspended in 0.04 N hydrochloric acid diluted in isopropanol. Samples were incubated for 5 min at RT. The absorbance was quantified by measuring the absorbance at 550 nm subtracted from the absorbance at 620 nm.

**Determination of Malondialdehyde (MDA) and Glutathione (GSH)**

MDA levels were measured using the Lipid Peroxidation Assay Kit (ab118970, Abcam). Cell lysates upon transfection of the HJURP shRNA vectors and scrambled shRNA plasmids were prepared according to the manufacturer's instructions. Samples and standards were incubated at 95°C for 1 hour, cooled to room temperature, and the absorbance was measured at 532 nm using a microplate reader (Synergy H1, BioTek Instruments). Glutathione (GSH) Level: GSH levels were determined using the Glutathione Assay Kit (CS0260, Sigma, St. Louis, MO, USA). Similarly, cell lysates were prepared, and the assay was conducted by adding the appropriate volume of reagent mix to each well of a 96-well plate. Samples and standards were incubated at room temperature for 30 minutes in the dark, and the fluorescence intensity was measured using a microplate reader (Synergy H1, BioTek Instruments) at 340 nm excitation and 412 nm emission wavelengths.

**Assessment of ROS**

After 48 hours of transfection with shRNA targeting HJURP or a scramble shRNA control, the breast cancer cells were seeded in 6-well plates. After 8 h, the cells were stained with 20 μM 2’,7’-dichlorofluorescein diacetates (Beyotime Biotechnology, Shanghai, China) in the dark at 37°C for 30 min. The ROS level in the cells was observed by a fluorescence microscope.

**RNA-seq analysis**

RNA-seq was performed to investigate the mRNA expression profiles after HJURP silencing in BT-549 and MDA-MB-231 cells. Three biological replicates were carried out at the LC-bio (Hangzhou, China) using HiSeq 2500 (Illumina, USA). Indexed samples were sequenced on an Illumina HiSeq 2500 in a single read mode. Differential gene expression was calculated applying the DESeq2 software (version 1.2.10). Fold change >1.5 were set as the cut-off values to select differentially expressed genes.

**Data mining**

Gene co-expression analysis was performed on 1084 invasive BCa tissue samples, the cancer genome atlas (TCGA) dataset (Breast Invasive Carcinoma, TCGA, PanCancer Atlas, 2018) and on 921 cancer cells lines, Cancer Cell Line Encyclopedia (Broad Institute and Novartis, 2019; Broad CCLE Portal.) using the c-BioPortal Cancer Genomics portal (http://www.cbioportal.org).

**Statistical Analysis**

SPSS 21.0 (IBM Corp., Armonk, NY, USA) was applied for data analysis. The measurement data are expressed in the form of mean ± standard deviation. Firstly, the normal distribution and variance homogeneity tests were conducted. If the data conformed to the normal distribution and homogeneity of variance, then the t test was used for comparison analysis between two groups. One-way or two-way analysis of variance (ANOVA) was used for comparison analysis among multiple groups, followed by Tukey’s multiple comparisons test. If the data did not conform to the normal distribution or homogeneity of variance, the rank sum test was carried out. The p <0.05 meant statistically significant.
